# Public discourse and wilful incommensurability: a case for attentive free speech

**DOI:** 10.3389/fsoc.2024.1178525

**Published:** 2024-05-22

**Authors:** Todd Smith, Benjamin W. Kelly

**Affiliations:** ^1^Association for Baha’i Studies, Ottawa, ON, Canada; ^2^Department of Psychology, Sociology, and Childhood Family Studies, Nipissing University, North Bay, ON, Canada

**Keywords:** attentive free speech, consultative epistemology, degraded free speech, liberal democracy, public discourse, safetyism, wilful incommensurability

## Abstract

Many thinkers lament the decline of liberal democracy. Some argue that, to rejuvenate it, we must think big. Thinking big involves generating new ideas about how to achieve an unprecedented level of social transformation aimed at cultivating solidarity, empowering citizen efficacy, and promoting the common good. We propose that fundamental to such a transformation must be a radical change in how people speak to one another. To this end, the primary objective of this paper is to offer a framework for understanding how speech currently erodes democratic engagement. The central idea is that much of speech today both reflects and perpetuates *a culture of wilful incommensurability*. The core features of this culture are *totalizing safetyism*, *expressive safetyism*, *dismissive intransigence*, and *polarized alienation*, all of which have been worsened by the current trajectory of social media. The result is that people are increasingly prone to engage in *degraded free speech*, which is characterized by a pervasive aversion to reach out, identify points of unity, benefit from diverse perspectives, and discover truth in all its potential complexity. In view of this diagnosis and the response of those who advocate for freedom of speech, a second objective of this paper is to introduce the concept of *attentive free speech*. Attentive free speech has similarities with civil discourse but is specifically characterized by discernment and thoughtfulness and is imbued with key dispositions such as courage, reverence, and love. We end by inviting future research into how such speech can promote the social and spiritual health of the public sphere and freedom itself at a practical level.

## 1 Context: the crisis of democracy

Over the past several years, many have worried that Western liberal democracy is at risk of collapsing (Goldberg, 2018; Stanley, 2018; Albright, 2019; Levitsky and Ziblatt, 2019; Applebaum, 2020; Ben-Chiat, 2020; Calhoun et al., 2022; Barnes et al., 2023). Some argue that for it to be resuscitated, it cannot continue as it has (McIntyre, 2023; Brooks, 2023b). It must, instead, transform in radical ways. This has become an urgent theme among many thinkers today in view of the exigent challenges confronting liberal democracies and humankind as a whole—challenges which are both multiplying and intensifying. As U.N. Secretary-General Guterres (2022) warned the leaders of the world on 20 September 2022, “Trust is crumbling. Inequalities are exploding. Our planet is burning… We cannot go on like this”.

In their book *Degenerations of Democracy*, Calhoun et al. (2022) address what they see as the crisis of liberal democracy head on. In Chapter 7 of this book, Calhoun and Taylor state, with forthright earnestness, that democracy is in severe jeopardy owing to “declining citizen efficacy, weakening local communities, fraying intergenerational bonds, evaporating small-scale economic opportunity, and eroding social ties that had once knit citizens together across lines of difference and fostered solidarity” (p. 261). Rising in its stead is a growing fascination with authoritarianism fueled by the proliferation of ignorant, delusional, and hyperbolic speech. Calhoun et al. maintain that the situation is especially urgent given the deeply intertwined environmental, economic, and social predicaments that now plague humanity. Time is running out on many fronts.

The degenerate condition into which democracy has fallen has become a pervasive topic. Headings of news articles such as “Arizona Republicans are making a case against the idea of democracy itself” (Zeeshan, 2022) are no longer surprising. Applebaum (2020) states that “[i]t is possible that we are already living through the twilight of democracy; that our civilization may already be heading for anarchy or tyranny, as the ancient philosophers and America’s founders once feared; that a new generation of *clercs*, the advocates of illiberal or authoritarian ideas, will come to power in the 21st century, just as they did in the twentieth; that their vision of the world, born of resentment, anger, or deep, messianic dreams, could triumph” (p. 185).^1^ She cautions that only together with carefully chosen friends “is it possible to avoid the temptations of the different forms of authoritarianism” (p. 188). Among these temptations is the now widespread attraction to reject truth. Pinker (2021) observes that “nothing from the cognitive psychology lab could have predicted QAnon” (p. 287). Similarly, McIntyre (2018) explains that “some now worry that we are well on our way to fulfilling that dark vision, where truth is the first casualty in the establishment of the authoritarian state” (p. 15). More recently he warns, “[o]nce truth dies, the end may come swiftly for American democracy… [W]e may even have further elections, but it will not really matter. If the truth killers succeed in using reality denial to undermine democracy, the next day we’ll wake up in an electoral dictatorship” (2023, pp. 4–5).

Associated with the casualty of truth is the discrediting of major institutions, which are increasingly deemed incapable of dealing with “a growing number of intractable public problems…that cannot be solved with technical fixes” (Longo and Shaffer, 2019, p. 14). These intractable problems include economic inequality, climate change, gun violence, pandemics, international conflict, religious conflict, war, demographic shifts, and persistent, systemic racism, sexism, misogyny, and other forms of bigotry. The time has therefore come to think big. Thinking big entails reimagining how to promote societal transformation and take seriously the social in social democracy (Calhoun et al., 2022), which includes our capacity to simply interact with each other on a daily basis. The *status quo* alternative, many agree, is unsustainable. As concluded by the American Academy of Arts and Sciences (2020) regarding its own nation, “we face these converging trends in a constitutional democracy that feels to many increasingly unresponsive, nonadaptive, and even antiquated” (p. 1).

Some also maintain there is reason not to lose hope. Brooks (2023a), for example, is optimistic because he finds opportunity in the dysfunction in the United States. He observes that “the story of America is a story of convulsion and reinvention,” that “as new problems become obvious, the culture shifts,” and “that open societies such as ours have an ability to adapt in a way that closed societies simply do not”. Similarly, the American Academy of Arts and Sciences (2020) believes that “a reinvention of our constitutional democracy remains entirely within reach” since “a superlative benefit of constitutional democracy, as articulated in both the Declaration of Independence and the Constitution, is that it is adaptable to new circumstances and unanticipated challenges” (p. 2). Yet, such thinkers recognize that monumental transformation is required if we—those of us who live in what are traditionally understood to be liberal, or Western-style democracies—are to surmount the degraded state in which democracy now languishes. We have become, as Brooks (2023b) also recognizes, “enmeshed in some sort of emotional, relational, and spiritual crisis”. The way out thus means thinking and acting in unprecedented ways.

## 2 Focus: the crisis of free speech

The main theme of this paper is that central to the crisis of liberal democracy is a crisis of free speech. On the one hand, the multiple problems we face make the need for civic reasoning urgent (Lee et al., 2021, p. 1). On the other hand, there is presently such polarization and ideological entrenchment that communication across groups results in little to no understanding (Barnes et al., 2023), thus stymying our capacity to come up with feasible, let alone sustainable, solutions (p. 225). Exacerbating the problem, civility, like truth and transparency, “has been hard hit”: now people increasingly feel free to say what they want, no matter how ignorant or hateful, with basically no regard for how others are affected (Calhoun et al., 2022, p. 280). There are certainly attempts to engage in reasonable discourse, cases where diplomacy wins out. But, on average, the cacophony of spiteful rhetoric is becoming increasingly oppressive, debilitating, and, in many cases, lethal. Public discourse has, in many respects, degraded into a calamitous state of specious recklessness.

In view of such observations, this paper has two objectives. The primary objective is to offer a framework for understanding the current condition of free speech in liberal democracies (there is no attempt to draw explicit comparisons to what are typically considered illiberal societies). This paper is chiefly diagnostic. The central idea is that much of speech today can be understood as both reflecting and perpetuating *a culture of wilful incommensurability*. By wilful incommensurability we mean not only a condition in which people, in their various factions, are incapable of seeing eye to eye, let alone discovering common ground. Worse, we mean a condition in which many purposely talk past one another—in which they even revel in talking past one another—and that they do so appealing to their right to free speech. We argue that the core features of this condition are (1) *totalizing safetyism*, which manifests as cultish submissiveness to the will and worldview of an authoritarian; (2) *expressive safetyism*, a condition in which refuge from perceived threatening speech is considered paramount; (3) *dismissive intransigence*, or the obsessive proclivity to defend a position notwithstanding the strength of countervailing evidence; and (4) *polarized alienation*, a state of being in which people live in different worlds, perpetually suspicious and fearful of one another. We also maintain that these features have been exacerbated by the omnipresence of social media.

There are certainly many exceptions to this condition. Compromises between rival groups are achieved here and there. Politicians do indeed cut favorable deals. One can additionally look to growing numbers of organizations that see benefit in creating inclusive cultures. This is a budding concern among many businesses, not-for-profit endeavors, and other institutions, as well as among communities that place a value on diversity. Individuals, moreover, vary in terms of how much they are caught up in the culture of wilful incommensurability. Many, for example, strive to be civil in their encounters and are generally successful. This is the case in both informal spaces and formal spaces, such as certain news programs. Others contribute to the pervasive incommensurability but do so for the most part unwittingly or unconsciously. Western nations also differ in terms of the degree to which they are consumed by this culture, as do different groups within these nations. Some, as a whole, are decidedly more afflicted by one or more of the four core features of wilful incommensurability than are others. Thus, by features, we also mean *tendencies* that are more or less pronounced across different liberal societies and contexts. Finally, most of the findings cited in this essay concern North America and particularly the United States; these findings cannot be freely generalized to other countries.

With such caveats in mind, our general thesis is that much of the public sphere is suffused with a penchant to decry or ridicule seemingly divergent perspectives. This fixation severely hampers our ability to function democratically or to deal productively with the myriad ominous challenges we now confront as citizens and as a human species. This essay is concerned with this widespread, public condition.

In view of this condition, the second objective of this essay is to provide a rational for inviting further research into the proposition that *the transformation now required crucially entails a radical change in how we speak to one another*. To this end, we consider some of the merits and challenges of the traditional approach to free speech, and then suggest that for speech to become truly free, it must evolve into what we call *attentive free speech*. We conclude by briefly outlining how this type of free speech can be understood to relate to, but also go beyond, current conceptions of civil discourse.

## 3 The culture of wilful incommensurability

As noted above, there are four core features of wilful incommensurability. They are closely related to each other. The first two are versions of *safetyism*, a societal condition in which safety, as Lukianoff and Haidt (2018) explain, is considered sacrosanct. Here, we distinguish *totalizing safetyism* and *expressive safetyism*. The third and fourth features of wilful incommensurability are *dismissive intransigence* and *polarized alienation*, respectively. We maintain that while people ostensibly value individual freedom and its expression so highly, owing to these four features of wilful incommensurability they are variously prone to engaging in what we call *degraded free speech*. Speech is degraded when it deprives itself of the opportunity to learn from diversity, find synergies between perspectives, and thus expand horizons of understanding.

### 3.1 Totalizing safetyism

The first feature of wilful incommensurability, *totalizing safetyism*, is characterized most predominantly by cultish submission to the will of a dominant authoritarian. Here, the masses acquiesce to the authoritarian’s construction of reality notwithstanding how demonstrably puerile, self-serving, and detached from reality it may be. This is partly because there is comfort in capitulating to his or her will—in escaping from the freedom to independently think and choose into the normalizing grip of this fascistic leader.

Fyodor Dostoevsky understood well the perils of this totalizing mentality. In *The Brothers Karamazov*, he has brother Ivan recount a story of the Grand Inquisitor confronting Jesus, whom he arrests because the latter’s presence among the people challenges his power. The gist of his monolog to Jesus, who remains quiet throughout, is that, whereas Jesus offers freedom of conscience and choice, the people ultimately seek refuge under the wings of authority because they find the prospect of freedom overwhelming. The Inquisitor says:

Man is tormented by no greater anxiety than to find someone to whom he can hand over quickly that gift of freedom with which that unhappy creature is born…. Did you forget that man prefers peace and even death to freedom of choice in the knowledge of good and evil? Nothing is more seductive for man than his freedom of conscience, but at the same time nothing is a greater torture. (Dostoyevsky, 1960, p. 129)

In the end, they “huddle close to [those in authority] in fear, as chicks to the hen” (p. 134).

Fromm (2004) builds on this theme, relating it specifically to the rise of Nazism and Hitler’s commanding sway over the masses in Germany. According to Fromm, “the frightened individual seeks for somebody or something to tie his self to; he cannot bear to be his own individual self any longer, and he tries frantically to get rid of it and to feel security again” (p. 130). Again, the longing for security is provoked by the torment that comes with freedom of choice. The irony is that in surrendering their individual consciences to the will of the authoritarian and the “truths” he or she espouses, the people convince themselves that they have finally tapped into actual truth and are thus free—or that they are fighting a sacred war to win freedom in line with this so-called truth.

The anguish is further driven by a longing to find sanctuary in totalizing unity and simplicity, safe from diversity and complexity, both of which involve a far nimbler mentality (Coleman, 2021). As Applebaum (2020) explains, the authoritarian disposition is a simple-minded one: those who embrace it recoil at the prospect of plurality. Instead, they aspire to something rudimentary that will make sense of the complexity. And the language they use both reflects and endorses this crudeness.

One of the simplest ideas is the concept of “the other”. Thinkers such as Greene (2014) point out that people are universally susceptible to drawing in-group versus out-group distinctions. According to Greene, the human brain—unless consciously checked through deliberate reasoning—gravitates toward this way of framing the world. Authoritarians seize upon this proclivity as a primary means to galvanize their followers. For example, a group might have legitimate concerns about how they have been perceived or treated by the political elite, the cultural left, or others. This feeling of grievance may also be exacerbated by the displacement, fragility, and injustice they sense and experience in modern society, tied to, for example, the destabilizing forces of globalization over the last number of decades (Sassen, 2014), the culture of contest that underpins the economic and political realms (Karlberg, 2004), the identity politics and cancel culture that have taken hold of many universities and other institutions (discussed below), and the growing crises pressing in upon the nation. Whatever the reason, the authoritarian capitalizes on these grievances and spins them into a full-fledged politics of resentment, rallying followers, as Fukuyama (2018) explains, “around the perception that the group’s dignity has been affronted” (p. 7). Harboring this resentment and stirred by their leader, the group naturally feels impelled to fight for public recognition of its dignity. It may go so far as to distinguish itself as a special, even hallowed, community that alone can address the crises before the population.

Thus, to secure power, the authoritarian finds it propitious to unify—actually, to homogenize—the group by identifying existential enemies and tapping into, fabricating, and circulating conspiracy theories that demonize these enemies, claiming, for example, that they are poisoning the blood of the nation—this appealing to blood purity being a common tactic (Hill, 2013). As noted above, one reason it is advantageous to advance such claims is because the simplicity of a conspiracy theory makes it emotionally attractive (Applebaum, 2020, p. 45). The same goes for the general concept of “us versus them”. Authoritarians therefore “do all they can to exacerbate strife” knowing that they “hold appeal when society is polarized, or divided into two opposing ideological camps” (Ben-Chiat, 2020, p. 8). Within this fraught milieu, the group coalesces around their leader with many among them arising to amplify and circulate his or her reductive yet malicious propaganda while also denigrating the free press or mainstream media as fake news.

Over time, with the incessant doubling down and ceaseless repetition of deceits, some public figures who initially held out find themselves drawn to support the cause. Perhaps they do so at first apprehensively. But many—including some who initially criticized the leader—eventually devote themselves ever-more doggedly to disparaging democratic values and independent institutions which they now claim to be obstructing the true path forward. They become overtly convinced (they may privately feel differently) that only the “true believers” in the falsehoods can achieve and retain the power and privilege they seek, and so style themselves as the real patriots with special access to the truth. Invariably, some even find themselves scrambling over one another to attack, or mortally threaten, disbelievers and to aggressively defend their leader who concomitantly finds it profitable to portray him or herself as a victim or martyr in the face of reproach.

The upshot is a relentless inundation of politicians, media personalities, influencers, social media mobs, and friends and family members, denying the most obvious of truths in support of an authoritarian who in turn has little qualms about ginning up hatred; whipping up followers into states of frenzy; targeting, intimidating, and vilifying opponents and innocents alike—even referring to them as vermin and related appalling terms; and fomenting discord and distrust of institutions and the mechanisms of justice at every turn. What eventually emerges are “dreams of ‘cleansing’ violence and an apocalyptic cultural clash” (Applebaum, 2020) as well as a growing number of “patriots” who arise to fulfill this dream with deadly consequences (p. 57). They, as Snyder (2017) explains, “trade real freedom for fake safety” (p. 100), which they nevertheless conceive of as exceptional, revolutionary, and fundamental for dealing with the crisis, or state of emergency, as their leader depicts it.

This leader on his or her part buys further into the flattering myths—some of which may have in fact originated among the masses—with swelling cultish zeal. He or she understands, as Klein (2023) observes, that “[c]lout is a calculus not of what you do, but of how much bulk you-ness there is in the world. You get clout by playing the victim. You get clout by victimizing others” (p. 106). You also get clout by inflaming supporters on social media and at political rallies by, in turn, unremittingly propagating bloated falsehoods, hyperbolic defamations, and baseless machinations—by propagating such degraded speech—and by promising to pardon allies, appoint only the loyal to key positions of power, and achieve retribution. And so it spirals: as the supporters become further inflamed and entrenched, their leader becomes increasingly brazen with the duplicities, hyperboles, and threats, and all the more fanatically committed to the self-serving myths and to slandering those who dare to refute them.

Applebaum (2020) asserts that “any political system built on logic and rationality was always at risk from an outburst of the irrational” (p. 15). The instigators behind this outburst are also prone to cheapening our understanding of history, distorting it into what she calls a “cartoon version” (p. 74) of itself. More generally, empirical facts dwindle in significance because they do not serve the will of the authoritarian and his or her sycophants, and because they demand engagement with reality—including other people’s lived realities and standpoints (Smith, 1989)—which can be perplexing and complex, thus challenging the believers’ sense of safety. Again, this sense of safety is intimately tied to simplicity, even if that simplicity does not actually make rational sense. In fact, the irrationality often helps. Facts are accordingly swapped for reductive slogans, myths, and the mysterious. On this point, Snyder (2017) states that “truth dies in four modes”, which, he explains, are open hostility to verifiable reality (treating lies as if they are facts); endless repetition (systematic use of a nickname); magical thinking (the open embrace of contradiction—the vote is always rigged, and you should vote for me anyway); and misplaced faith (I alone can solve it) (p. 66). These modes, moreover, are all part of what McIntyre calls the post-truth playbook, which, as he puts it, goes like this:

[A]ttack the truth tellers, lie about anything and everything, manufacture disinformation, encourage distrust and polarization, create confusion and cynicism, then claim that the truth is available only from the leader himself. The goal is not merely to get people to believe any particular false claim, but to so demoralize them with a tsunami of falsehoods that they begin to give up on the idea that truth can be known at all, outside a political context. (2023, p. 3)

In other words, truth is treated with contempt: it means nothing unless it serves the interests or the whims of the demagogue, party, or faction. If it does not, it must be swept aside in favor of more convenient constructions, which are then reified as truth—so long, that is, as they remain personally, politically, or factionally useful.

Writing in the late nineteenth century, Bahá’u’lláh (2017) warned of this enthused abandonment of reality: “Witness how they have entangled themselves with their idle fancies and vain imaginations… They are themselves the victims of what their own hearts have devised, and yet they perceive it not” (p. 231). Today we witness a similar phenomenon in different parts of the world: many, seduced by totalising safetyism, are entangled by their own quixotic, vainglorious fascinations. As such, they harden the walls of incommensurability between “us” and “them,” degrading speech in conformity with the idle fancies of the authoritarian. They embrace such conformity—they find refuge in it—because it ostensibly addresses their sense of rootlessness, justifies the resentment they feel toward the perceived other, and thus provides a measure of apparent coherence to their world. Like the Grand Inquisitor’s chicks, they huddle close to their leader no matter how deluded, illogical, self-indulgent, and vitriolic—how degraded—his or her speech may be. They find consolation, meaning, and purpose in such speech and so enthusiastically propagate it while more dubious others end up rationalizing it for the sake of expediency and for fear of otherwise being labeled and attacked as disloyal. Yet others do in fact resist but are then indeed labeled and attacked.

### 3.2 Expressive safetyism

The second manifestation safetyism, *expressive safetyism*, is associated with the rise of safe spaces and other emotional security measures meant to protect individuals against uncomfortable—often portrayed as violent—speech. A related phenomenon is the rise of self-censoring (and censoring) caused by the growing prospect of being persecuted should something be said that contravenes what is presently touted by an insistent, vocal group as the correct way to think and speak. As we point out below, we admittedly have more sympathy for this manifestation of safetyism over totalizing safetyism. However, we also argue that both manifestations purvey false safety while perpetuating the climate of wilful incommensurability—that they both, consequently, fan the flame of degraded free speech.

Expressive safetyism is a growing feature of a type of liberalism that is focused on group-identity-based politics comingled with an accent on authentic self-actualization. One implication of this development has been the rise of intolerance toward voices that are perceived to inadequately reflect the latest progressive views. This trend has spawned a paradox for progressive liberalism: in their effort to uplift certain forms of difference, some liberal advocates resort to the illiberal practice of silencing other forms of difference, specifically as it relates to diversity of speech and conscience. Doyle (2021) explains that “[a] new identity-based conceptualization of ‘social justice’ brought with it a mistrust of unfettered speech” (p. 7). What has emerged in its stead is what he calls “the well-intentioned authoritarian” (p. 7) or “the arbiters of permissible speech and thought” (p. 9), who, according to Fukuyama (2018), “legitimate only certain identities while ignoring or denigrating others” (p. 119). This raises serious dilemmas, one of which is the question: Who gets to decide who the arbiters of permissible speech are? And, as Doyle warns, if we allow such arbiters to exist now, no matter how righteous their views may seem, does not making such an allowance pave the way for a ruthless government to step in down the road and claim that it is uniquely qualified for the role?

As noted below in this section, some speech can undeniably cause harm—particularly speech that disrespects human rights or human dignity. Yet, for all the good intentions behind it, the excessive arbitration of permissible speech can be patronizing and contribute directly to infantilization and fragility. This dynamic has become a widespread concern in institutions such as colleges and universities, where freedom of speech has traditionally been viewed as essential to the generation of knowledge and the cultivation of minds. Through what Lukianoff and Haidt (2018) call concept creep, the idea of what it means to be unsafe has been expanded beyond the risk of, say, physical harm to include students’ subjective feelings of discomfort with alien ideas. They explain that a cult of safety has emerged, “an obsession with eliminating threats…[which] deprives young people of the experiences that their antifragile minds need, thereby making them more fragile, anxious, and prone to seeing themselves as victims” (p. 32).

While well-intentioned, this overprotection, in Lukianoff and Haidt’s view, erodes the capacity of students to withstand, let alone profit from, challenges. It diminishes what Taleb (2012) calls their antifragility. In place of developing resilience and their critical thinking skills, students learn instead to cognitively distort reality in line with their social-historical affinities and commitments by exaggerating and dichotomizing what, on first encounter, they perceive as threats, and allowing their initial, negative emotional responses to these threats to hold sway. So much that happens outside the safe space is treated with apprehension and anger for fear that it may provoke mental health issues. Han (2021) identifies a similar phenomenon on a larger scale, stating that society is ruled today by a universal *algophobia*, which is a generalized fear of pain, and that this fear has resulted in a *permanent anesthesia* (p. 1). Mukhopadhyay (2016) in turn writes that “[n]ot all suffering is illness; some suffering is political” (p. 23) and argues that problems produced “by social norms, arrangements, and representational regimes…are pathologized” (Bisaillon, 2018, p. 104). It seems that the suffering—the discomfort and anxiety—that accompanies a generalized fear of seemingly alien ideas has been socially constructed as one such problem. Trigger warnings consequently abound.

Many go on to argue that this culture stymies freedom of speech. Douglas et al. (2021) for example, reference a national survey that found 63% of students agreeing that the climate on campus “prevents some people from saying things they believe because others might find it offensive” and that it is common for students across the political spectrum and faculty to self-censor (p. 12). They also point to other surveys that find a significant minority of students are willing to shut down speech they find objectionable. More recently, the Foundation for Individual Rights and Expression (FIRE) and College Pulse found that, among 55,000 students surveyed across 248 colleges, 56% of students “expressed worry about damaging their reputation because of someone misunderstanding what they have said or done” (Stevens, 2023, p. 2), which is slightly down from the previous year, and that 63% of students say it is acceptable to “[shout] down a speaker to prevent them from speaking on campus” (ibid., p. 35).^2^

Many professors also learn to self-censor because they worry about saying or writing something that may not conform to the received wisdom of the moment or the lived experience of one or more students in the classroom. It is not that this wisdom or lived experience is not valid; frequently it is. Indeed, oftentimes it plays an important part in the search for truth. Rather, the problem is that sometimes there is much more to the picture that warrants consideration. When one or more people’s lived experience is taken as the only valid information, thereby shutting down different perspectives that may have some validity of their own, seeming offenses can generate severely disproportionate reactions. Professors, for example, can be targeted as villains and can lose their jobs for minor infractions. Lukianoff and Haidt provide telling examples of such reactions in their book (see, for example, Chapter 3). Douglas et al. (2021) helpfully summarize the present state of affairs:

There is overwhelming survey research and other evidence that the intellectual climate on many college and university campuses is being constrained. Faculty are deterred from exploring certain subjects and expressing candid opinions even off campus; students are self-censoring; outside speakers are disinvited and events are being canceled. Social media has become a megaphone that amplifies campus controversies, increasing their intensity and visibility, compressing time frames for a leadership response, and leading to investigation and sanctioning of faculty and students. The traditional understanding of free speech as a liberalizing force is itself being called into question. (p. 6)

A central issue here is that the feeling of being offended often takes precedence over analytical thinking. This feeling is deemed to provide sufficient grounds for silencing and thus delegitimizing an apparently inauspicious opinion or argument. And because the opinion provokes anger, the delegitimization is habitually accomplished through verbal attacks, *ad hominem* arguments often punishingly rehearsed over social media, and angry mobs shouting down speakers and pressing university administrators to sanction “offending” professors. Meanwhile, colleagues of the targets publicly hold their tongues, frightened that they will be depicted as offenders themselves. Reason succumbs to emotion and fear all around.

Rauch (2021) and many others cited in this essay remind us that universities are meant to be spaces in which minds are challenged to think beyond what may be comfortable to think. This is the position recently taken by Cornell President Martha Pollock, who vetoed her school’s student government’s decision “to put trigger warnings on the school’s syllabus for any course containing content that might be viewed as ‘traumatic’” (Avlon, 2023). In her letter to the provost explaining her veto, she states that “[l]earning to engage with difficult and challenging ideas is a core part of a university education” (ibid.). More generally, being able to think well requires being exposed to viewpoint diversity.

But such diversity withers in this climate of expressive safetyism, which is now impacting the culture of many universities. These institutions are “at the forefront of finding ways to suppress opinions” (Pinker, 2021, p. 43) and are, notwithstanding their emphasis on diversity, becoming “increasingly ideologically conformist” (Douglas et al., 2021, p. 11). Many argue that this is extremely detrimental to thinking and to society in general, because “when an institution punishes internal dissent, it shoots darts into its own brain” (Haidt, 2022). Doyle (2021) agrees, claiming, “[t]he costs to the intellectual wellbeing of society can hardly be overestimated” (p. 61), and that “the eventual impact of our collective silence will be an enervated and infantile culture” (p. 63). Many also argue the infantilization has in fact already happened and that it is deleteriously affecting classroom dynamics across North America. Douglas et al. (2021) draw this conclusion, stating in addition that, “[t]he chilling of campus speech is having effects beyond the borders of the campus” (p. 6). Moreover, “the inhibition of campus speech” not only fails to alleviate political polarization, it also degrades “the civic mission of higher education, which is to maintain our pluralistic democracy by preparing students for civic participation as independent thinkers who can tolerate contrary viewpoints and work constructively with those with whom they have principled disagreements” (ibid.).^3^

It should be noted here that there have been other moments in history when free expression and academic freedom were similarly challenged and constrained by propaganda. For example, in the early 20th century in the United States, academic fields became increasingly professionalized (Douglas et al., 2021, p. 7). This led to a transformation in disciplinary research which many considered objectionable and so sought to sanction it. Moreover, the turmoil during Civil Rights and Vietnam War era led to a reconsideration of the rights of student protestors to express themselves. In response, both these moments, among others, prompted the drafting of influential statements on academic freedom. Given these historical developments, Douglas et al. offer the following optimistic outlook: “Ours is a similarly powerful moment of political and social change and of new trends in higher education. Looking back on the successes of these previous efforts to find new ways to uphold free expression values, we are confident that colleges can renew their approach to fostering free expression and open inquiry” (ibid.).

Now an understandable objection to these observations is that, in many respects, expressive safetyism is a natural reaction to the fact that certain voices have been systematically marginalized and so deserve to be protected from hateful speech that contravenes their dignity as persons. As discussed below, an equally legitimate concern is that certain speech can indeed cause harm to individuals who identify as minorities. This is buttressed by the fact that, recently, some politicians have undertaken to suppress freedom of speech by seeking to remove or whitewash the teaching of certain subjects to avoid dealing with narratives that highlight the painful history of slavery or the genocide of Indigenous populations (Benson, 2022; Brown, 2024). For such reasons, if we, the authors, had to choose between the two forms of safetyism, we would readily make the case that expressive safetyism as manifested in universities and other institutions is far more understandable and less objectionable than totalizing safetyism.

Yet, it is becoming increasingly evident that the problem of marginalization is not solved, as Mitchell (2022) puts it, by “[g]enuflecting to individuals solely based on their socialized identities or personal stories”. Certainly, and as already pointed out, lived experience can be a valid source of insight into the nature of a given issue or phenomenon. Specifically, those who have been marginalized often have “a distinctive range of experience and set of conceptual resources” that can disclose realities and unveil limiting assumptions that would otherwise be missed or taken for granted (Wylie, 2015, p. 79). This has become clear at least since Du Bois (1993) introduced the concept of “double consciousness”—an ability of African Americans to understand their conditions and society from both the dominant and their own racially oppressed perspectives. Accordingly, it would be impulsive, and thus counterproductive, to pre-emptively discount situated knowledge in favor of more dominant views.

The solution, however, does not seem to be a form of deference politics. This is because such genuflecting, Mitchell goes on, is also “a form charity that weakens the individual and the collective”. In other words, “[w]e infantilize members of historically marginalized or oppressed groups by seeking to placate or pander instead of being in a right relationship, which requires struggle, debate, disagreement, and hard work”. As Táíwò (2022) explains, politics of this sort “asks the traumatized to shoulder burdens alone that we ought to share collectively, lifting them up onto a pedestal in order to hide below them” (p. 117). He specifically makes this point with respect to his own lived experience:

That I have experienced my share of traumatic experiences, have survived abuse of various kinds, have faced near death from accidental circumstances and from violence…is not a card to play in gamified social interaction or a weapon to wield in battles over prestige. It is not what gives me a special right to speak, to evaluate, or to decide for a group. It is a concrete, experiential manifestation of the vulnerability that connects me to most of the people on this earth. It comes between me and other people not as a wall, but as a bridge. (2022, p. 118)

We agree with this assessment but suggest toward the end of our essay that the most productive way to truly learn from diversity while overcoming marginalization—a key objective of expressive safetyism—is to practice attentive free speech. This entails moving beyond safetyism in both its forms, neither of which is ultimately conducive to building the necessary bridges that enable fruitful, empowering, dialog. Neither form of safetyism encourages rational, critical, let alone courageous thinking in the face of diversity. Instead, both forms valorize reactive, negative, emotional responses, such as grievance, contempt, and fear.^4^ Whether or not a negative emotional response is sincere, it is privileged over reasonable deliberation, pre-emptively nullifying the potential merit of alternative points of view. It can thus inhibit others from sharing their authentic views (and emotions) for fear, on their part, of reprisal.^5^

### 3.3 Dismissive intransigence

While totalizing safetyism and expressive safetyism are distinguishable in many ways, they both help to perpetuate wilful incommensurability and degraded free speech. This is partly because they both contribute to, and overlap with, the next two features of wilful incommensurability: dismissive intransigence and polarized alienation.

By dismissive intransigence is meant an approach to reasoning where people vigorously, even obsessively, vindicate their own opinions or beliefs notwithstanding the strength of the countervailing evidence exposed to them. They do whatever they can to affirm their way of thinking while purposefully going out of their way to reject or denigrate that which challenges their thinking and its sources.

We are naturally inclined to confirm what we take, or want, to be true. We all have the subconscious tendency to select and interpret evidence in ways that support our beliefs. This is called confirmation bias. More than that, we are also prone to engage in motivated reasoning. Sometimes we actively ignore facts, select some facts over others, and interpret the facts we do select in accordance with our background assumptions and preconceptions. Owing to media bubbles and related factors, some of us think, as Rauch (2021) puts it, that we are “making rational decisions based on what appears to be solid evidence—unaware that we are trapped in a closed loop” (p. 246). At the same time, we often consciously choose which loops in which to become trapped. We are, as Coleman (2021) observes, “highly emotional, esteem-thirsty, cognitive misers” (p. 28).

Confirmation bias and motivated reasoning are well-known phenomena. However, the problem has swollen with the rise of safetyism. Now we (recalling the variation among us) tend towards remaining deliberately ignorant.^6^ An example is that “[d]eniers and other ideologues routinely embrace an obscenely high standard of doubt toward facts that they do not want to believe, alongside complete credulity toward any facts that fit with their agenda” (McIntyre, 2018, p. 18). They “follow the same flawed reasoning strategy,” namely, cherry-picking data, believing in conspiracy theories, engaging in illogical reasoning, relying on fake experts (and denigrating real experts), and having impossible expectations for what the other side must produce for evidence (McIntyre, 2023, p. 15). And this is now done bald-facedly. McIntyre goes on: “Apparently one does not even have to hide one’s strategy anymore. In an environment in which partisanship can be assumed, and it is often enough to ‘pick a team’ rather than look at the evidence, misinformation can be spread in the open and fact-checking can be disparaged” (2018, p. 30).

In other words, motivated reasoning has now ballooned into the duplicitous practice of intentionally distorting facts, flooding media with rival constructed facts (see Section 4 below), and propagandizing corresponding “interpretations” to suit personal or partisan objectives, often hateful ones. There is little interest, if any, in entertaining facts that challenge one’s worldview. People see what they want to see no matter how compelling the countervailing evidence may be—no matter, for example, how extensive or blatant the evidence that a candidate for office is only in it for him or herself or that climate change is real. As McIntyre puts it, “the problem today is that the same toxic form of reasoning has now metastasized from science denial to reality denial” (2023, p. 17). This form of reasoning we call dismissive intransigence.

A basic characteristic of dismissive intransigence is simple-minded thinking. This involves unwarranted binary thinking (some binaries may be warranted) and various forms of unforgiving reductionism, including rigid categorization. Ripley (2021) explains that the main objective of such approaches to categorization is to “[wash] out all the details and contradictions so we can draw a crystalline partition between good and evil, right and wrong” (p. 97). At one level, we employ these crude strategies because it is convenient to do so—it saves us the bother of having to really think about the way things are. At a deeper level, such strategies help us to feel better about ourselves, but often at the expense of others. Grappling with complexities and convergences, contrarily, makes us feel insecure and anxious. It also takes work to situate our thoughts within a coherent framework of understanding. So we gravitate toward taking reductionist short cuts. A good example of this tendency is Mitchell’s characterization of virtue signaling: “It’s easier to use language and cultural references that signify an ideological inclination than to actually study and practice a particular framework. However, such loose ideological signaling can lead to incoherence. This practice can devolve legitimate frameworks, concepts, and language into tools for individuals to virtue signal or provide weight to an argument that does not stand on its own premises” (2022).

Again, this tendency has significantly worsened in the current political climate. A major reason for this is the increasing deployment of disinformation (McQuade, 2024), which is used to undermine confidence in facts that contravene the worldview or interests of the perpetrator as well as in the sincerity of those who draw attention to such facts: “The genius of disinformation is that it does not just get you to believe a falsehood, but to distrust (and sometimes even hate) anyone who does not also believe this same falsehood” (McIntyre, 2023, p. 24). By cultivating doubt—through the constant repetition of lies, “whataboutism,” and other like strategies—about a particular truth while also slandering those who stand for it, the disinformer succeeds in propagating the reality he or she prefers. In fact, “distrust, and not just doubt, is the prime objective of a denialist campaign. Mere doubt can be overcome with evidence, but distrust cannot” (McIntyre, 2023, p. 25). The disinformer’s joint objective, therefore, is to get the audience to both believe in the false information and feel that the “other side” is the enemy (McIntyre, 2023, p. 97). It is to exacerbate the polarization (McQuade, 2024).

This constructed reality can also lead to a state of resignation, because, with the flurry of falsehoods swirling around, little can be made sense of anymore. The disinformation disorients and deludes, creating chaos and confusion that leaves people exhausted and cynical about politics (Bazelon, 2022, p. 42). On the other hand, it helps to delineate who is opposed to whom, and so reifies the polarization. It carves out a basic us-or-them reality that many come to accept with little question, let alone serious scrutiny, and so is at least simple and clear in this respect.

This brings us to the fourth, overlapping feature of the culture of wilful incommensurability.

### 3.4 Polarized alienation

Polarization is one of the most urgent problems we face today (Coleman, 2021; Lee et al., 2021; Boxell et al., 2022). We hear assertions from thinkers and leaders all the time that we are “are riven by affective polarization and divisive stereotypes about our political opposites” (Douglas et al., 2021, p. 10) and that we are living in different realities, constantly demonizing and fearful of each other.^7^

Arendt (1977), referring to Immanuel Kant, states: “The power of judgment rests on a potential agreement with others, and the thinking process…finds itself always and primarily, even if I am quite alone in making up my mind, in an anticipated communication with others with whom I know I must finally come to some agreement” (p. 217). She then observes that “this enlarged way of thinking…needs the presence of others ‘in whose place’ it must think, whose perspectives it must take into consideration, and without whom it never has the opportunity to operate at all” (ibid.). In other words, intersubjectivity is vital for thinking to be all it can truly be. But both forms safetyism and dismissive intransigence—the first three overlapping features of the culture of wilful incommensurability—are antithetical to achieving this “enlarged mentality” (totalizing safetyism being more problematic here than expressive safetyism). Instead, they lure us into myopia, into the dogmatic conviction that our beliefs are increasingly vindicated the more they conflict with the beliefs of others. Sympathetic thinking is usurped by motivated hate, fear, and disdain for these others. We end up in a state of what Ripley (2021) labels “high conflict,” which she defines as an us-versus-them conflict that is self-perpetuating and all-consuming.^8^ Its main characteristic is stagnation, where, in fact, conflict itself becomes the goal and where we find purpose in demonizing others. We are also inclined to caricature these others because “[i]t is easier to dismiss and demean a cartoon villain” (Ripley, 2021, p. 134).

On this point, it is well known that the end has often been used to justify the means. However, we now face the additional quandary that the means (discord) is often used to justify the end (more discord). While the quest for utopia may have inspired conflict many times in the past, and still does, now conflict also vindicates more conflict as an ultimate end. In the first case, there is no consistency between means and ends. In the latter case, the consistency is conflict. Both cases subvert the enlarged way of thinking that Arendt champions, thus creating a condition that solidifies polarization and hence alienation in at least three ways.

In the first place, this condition obviously leads to our alienation from each other, especially since so many of us feel humiliated by, or resentful of—and thus learn to hate—those outside our groups. Ripley (2021) explains that “[i]f humiliation is the nuclear bomb of emotions, hatred is the radioactive fallout. That’s because hatred assumes the enemy is immutable. If the enemy will always be evil, there is no reason to ever consider any creative solutions to the conflict” (p. 131).

In this condition, we are also alienated both from reality and from ourselves. We are alienated from reality because, as we encamp ourselves within our respective worldviews, we cut ourselves off from the possibility of benefiting from insights that may be relevant to understanding it more fully and constructively. We are alienated from ourselves because, in our efforts to shield ourselves from challenge and otherness, we run from our inherent freedom and the choice to think outside our gratuitous prejudices. Uttering degraded speech toward each other, we perpetuate our fragility and undermine our capacity to creatively tackle the encroaching existential problems of the world, much less foster the democratic solidarity necessary to flourish both individually and collectively. Worse, we remain ensnared within a web of parochial delusions or self-referential fantasies, thus impeding our own potential to become all we can truly be. We flounder in a state of degraded freedom.

### 3.5 Summary

The central thesis of this paper is that society is generally afflicted by a culture of wilful incommensurability, recognizing that this condition is far more pronounced in some contexts than in others (recalling the caveats outlined in Section 2). There are four core, overlapping features, or tendencies, of this culture: both totalizing and expressive safetyism, dismissive intransigence, and polarized alienation. Owing to these features, we are largely in a condition in which we—or many among us in our liberal public sphere—are inclined to obstinately talk past one another. Encased in our ideological paradigms or factional worldviews, we make little or no attempt to see eye to eye let alone learn from each other. Instead, we are prone to engaging in degraded speech because we find security and meaning—counterproductive and obdurate as it may be—in perpetuating the impasses we have erected between ourselves.

In coming to this conclusion, it is important to acknowledge that incommensurability is a complicated subject and refers to different although related conditions. In one of its more dramatic formulations, it stands for the following: different paradigms or worldviews are unintelligible to each other; meaningful communication between them is therefore untenable. To effectively communicate across paradigms is in the last analysis to reject your own. Like a gestalt switch, it is to leap into a wholly new mode of thinking and being. In this politicized environment, it is to defect to the world of the other.

There are admittedly problems with this formulation of incommensurability. It is also not necessarily the one that Kuhn (2012)—who arguably came up with the term while examining how the scientific community operates—promotes, nor, for that matter, Feyerabend (1993), another well-known philosopher of science who emphasized the contingency of scientific methods. There are also other philosophers who basically reject the idea altogether. Popper (1994), for example, refers to the “myth of the framework,” which, as Bernstein (2010) helpfully explains, is “the myth that ‘we are prisoners caught in the framework of our theories; our past expectations; our language,’ and that we are so locked into these frameworks that we cannot communicate with those encased in ‘radically different’ frameworks or paradigms” (p. 54).

Theoretically, we, the authors, agree with this position. Paradigms may be integrated, articulated, self-justifying, but they are not ineluctably shut off from each other. And their protagonists are not necessarily wrapped up within them. They may be predisposed to the internal logics of their paradigms; they may even consciously venerate these internal logics. But these paradigms are not inescapably ensnaring. It is possible to reach out and constructively explore that which seems foreign.

The problem is that we—or, again, many among us in the public sphere—are reluctant if not loath to seriously entertain the merits of other paradigmatic views. We are put off by even the prospect of considering their merits, intentionally entangled by our biases and prejudices and so uninspired to seek solidarity beyond the group (in fact, quite the opposite). We flout that which is potentially conducive to both individual and collective betterment because we spurn Mill’s (1978) admonition that we should continually subject our conceptions of truth to the scrutiny of diverse others lest we fall into dogmatism (no matter how correct our conceptions may be) and thus fail to see potentially veritable facets of the truth. Far from achieving shared understanding, we are motivated to undercut the perspectives of others and emulsify our own opinions (if not always our thoughts) in accordance with the pronouncements of the influential. Admittedly, on good days, we arrive at compromises with those we consider alien to us, but rarely do we do so in a way that does not agitate the already festering resentment of one or another faction or party. To avoid the agitation, the risk, we are drawn into safeguarding—we are motivated to preserve—the walls of incommensurability. And we end up doing so wilfully.

## 4 Social media as an exacerbating factor

This passionate, wilful thoughtlessness has become especially pronounced now that social media has taken hold of so much of our lives. Social media, particularly over the last decade and a half or so, has played an outsized role in manipulating thinking, hardening ignorance, facilitating bullying and toxicity, and fortifying the barriers to inter-party and inter-factional discourse (Harris, 2017; Schirch, 2021; Safi, 2023; Quinelato, 2024; Recuero, 2024). Our point is not that social media is inherently destructive. The degree to which it is, or is not, is a subject for another paper. In brief, there are good arguments to be made that it has had beneficial consequences, such as creating new connections, fostering discourse, giving voice to marginalized voices, and holding the powerful accountable (Schirch, 2021; Ceresney et al., 2022, p. xxiii).

Rather, what we are saying is that, in its current form, social media contributes profoundly to the degradation of democratic engagement and our capacity to cultivate solidarity across groups. We are now “in an era when rational dialog and debate had been abandoned for the high of in-your-face confrontation, with social media as an accelerant” (Walter Kimbrough in Harris, 2017). It thus exacerbates the culture of wilful incommensurability and the accompanying degradation of free speech.

Social media has, for example, introduced tools such as the “Like” and “Repost” buttons which enable opinions and rumors to be disseminated and presumably legitimized with unprecedented speed and with little or no accountability or reflection. It could be argued that many have become both seduced and tyrannized by this quintessence of reductionism. In any case, we now face “avalanches of misinformation, some of it coming from ignorance and some from untraceable, malevolent sources, drowning out accurate, fact-based information in oceans of misdirection, misinformation, and lies” (Minow et al., 2022, p. 285). In addition, many have been manipulated by social media algorithms which train them to click on, upvote, or “like” news, videos, or hastily crafted, reactive, often spiteful comments that insidiously lead them down mutually antagonistic ideological rabbit holes. In so doing, these platforms open them up to manipulation by trolls, extremist ideological provocateurs, and others who have no investment in the truth but who readily spread disinformation and share posts with “moral-emotional content” (Fisher, 2022, p. 156) or laced with incendiary, racist, anti-Semitic, Islamophobic (and the like) rhetoric, all meant to trigger emotions and discordant instincts simply because outrage is rewarded with more clicks, likes, and reposts—a new currency of discourse (Douglas et al., 2021, p. 9; Schirch, 2021; Ceresney et al., 2022; McIntyre, 2023).

This conjoining of the trivial, the reductive, the impulsive, the egoistic, the emotional, the moralizing, the polarizing, and the instantaneously viral is a new phenomenon. Now almost anyone can spread hyperbole, disinformation, and ignorant denunciations—this person’s phobic, that person’s a traitor—at unprecedented speeds, all easily packaged as newsworthy and apparently reliable (Ceresney et al., 2022; Kramer, 2022) with a predictable percentage of consumers falling for this “news” notwithstanding subsequent information that may correct it (McIntyre, 2023, p. 83). This situation has thus amplified the erosion of trust in institutions, the stoking of resentment between groups, and hence both forms of safetyism, dismissive intransigence, and polarized alienation that are at the core of the culture of wilful incommensurability.

Haidt (2022) quotes James Madison who worried that “people are so prone to factionalism that ‘where no substantial occasion presents itself, the most frivolous and fanciful distinctions have been sufficient to kindle their unfriendly passions and excite their most violent conflicts’”. This proclivity is now intensified by the fact that, on social media, users are drawn to the “the loudest and most obnoxious marginal voices” (Doyle, 2021, p. 29). It thus motivates users to amplify sentiments and political content that flatters their groups while disparaging and alienating those on the outside (Fisher, 2022).

The proclivity is further magnified by the fact that, again, basically any one of us can summarily castigate anyone else for uttering an “offensive” remark no matter how trivial the offense may be. We are simultaneously emboldened to administer such “justice” because of the dopamine hits we amass in so doing, and because we feel unrestrained by regular social cues and checks. This adds up to a state of fretful stupefaction which is sustained because we abandon critical thinking in favor of rationalizing the received opinions of the partisan mob. We descend into Alexis de Tocqueville’s and John Stuart Mill’s shared nightmare: tyranny of the (perceived) majority. (Perceived, because the “majority” is often just a very loud, social-media-prominent, minority.) And all this, importantly, is to say nothing of the violence and mental health issues that result as a consequence (Dhir et al., 2018; Hussain and Griffiths, 2018; Abbasi and Drouin, 2019; Baltaci, 2019; Longstreet et al., 2019; Fisher, 2022; Haidt, 2024; Helvich et al., 2024).

The upshot is a constant “manipulation of our emotions… Anger and hate are literally shaping who we will become as a people. It’s pumping toxic sludge through us” (Ressa, 2022, p. 264). Through social media, we become more alienated from each other than would otherwise be the case because we can more efficiently and expansively repudiate one another’s universes and the potential insights harbored within them. We also become more alienated from ourselves because we do not personally benefit from these insights and/or because we increasingly find safety in different worlds that are largely fashioned by the influential people in those worlds. We thereby rob ourselves of the opportunity to cultivate beneficial relations with others who can help us grow and mature by attuning us to other perspectives and their potential advantages. We succumb to simplistic thinking and the impulsive, single-minded pursuit of dopamine-centric validation secured by an outpouring of online schismatic praise. Consistent with the state of dismissive intransigence, there is little patience for good arguments and the independent investigation of truth, dominated as we are by the reductionist, moral-emotional content (often, outrage) pushed by social-media systems of recommendation. There is additionally little patience for reading well thought-out essays, let alone scholarly articles, when one-dimensional opinion pieces and information can be so easily sourced. In short, we further shelter ourselves within the quagmire of wilful incommensurability, further corrupt our already degraded speech, and thus further deprive ourselves of the liberty to enlarge our mentalities and transcend our degraded freedom.

## 5 The centrality of freedom of speech

Observations such as those outlined so far in this essay have recently led to a flurry of articles and books extolling freedom of speech. Of particular concern is that speech is being degraded by the rise of what we have termed totalizing and expressive safetyism, dismissive intransigence, and polarized alienation—the four core features of wilful incommensurability—all of which has been exacerbated by the current trajectory of social media. Compelling arguments are made on behalf of free speech, many of which, if not all, link it to the vitality of democracy and society. Two recent examples are books by Doyle (2021) and Rauch (2021).

One of Doyle’s main arguments is that “preventing individuals from expressing themselves as they see fit represents a far greater menace to social cohesion” (p. 10) than does allowing bad, even hurtful, ideas to propagate. One reason for this is that silencing such ideas only makes martyrs out the purveyors of those ideas, which in turn only bolsters their respective causes. Doyle says that “in censoring the abusive individual we lose the opportunity to expose the iniquity of their beliefs through public admonition” (p. 89). We also lose the opportunity—back to Mill again—to test our own ideas for their fecundity. The reason we engage in argument, according to Doyle, is “to refine our point of view, to challenge our certainties, and to persuade others when we feel they are misguided” (p. 89). And this is best accomplished by acknowledging that there may be some element of truth, no matter how faint, even among those views we find especially repulsive. If, instead, we censure speech because we worry it facilitates the dissemination of bad ideas—if we rashly decide which ideas are beyond the pale—we allow our own prejudices to prevail unchallenged. Living truths thus become dead dogmas, the prevalence of which dulls freedom itself. Thus, for Doyle, “[d]efending free speech means defending the rights of those whose speech we despise” (p. 20). To this end, he quotes Thomas Paine, who warns: “He that would make his own liberty secure, must guard even his enemy from oppression; for if he violates this duty, he establishes a precedent that will reach to himself” (p. 21).

Rauch (2021) agrees. His arguments for the benefits of free speech are far-reaching and we cannot do them justice here. Suffice it to say that among his many themes is an elaboration on the metaphor of the marketplace of ideas, which, he explains, “draws upon pro-speech arguments from Milton and Mill to the present day” and “makes them instantly intuitive in today’s consumerist world” (p. 120). In line with this metaphor, he argues that “[i]deas are like cereals in the grocery store, and we are like shoppers, and competition drives the market toward more and better products!” (p. 120). But, as he goes on to explain, this metaphor only goes so far because it leaves out the important fact that “ideas do not sell, exchange, or compete all by themselves” (p. 120). Rather, the central mechanism for the exchange of ideas, and hence for the generation of knowledge more generally, is persuasion and nonstop negotiation between competing participants in the exchange. There is, consequently, no room for silencing others (there is no final say on what’s right or true) or for dominating them (there is no personal authority). On the contrary, if you “cannot convince others you are right…then you need to try some other proposition or some other approach” (p. 121). To participate in the marketplace of ideas is invariably to adapt, acknowledging that you are “in the business of contending, persuading, compromising” (p. 121). This is because, by “expos[ing] your ideas to peer review and public debate…[y]ou will be forced to adjust your thinking and your strategy, and as the process is repeated millions of times a day across the reality-based network, the whole system becomes a dynamic web of mutual persuasion: critical persuasion, so to speak, a social process of continuously comparing notes and spotting errors and proposing solutions” (p. 121).

Vital to this arena of persuasion is the ability of individuals to “accept the legitimacy of criticism and hold themselves accountable to it” (p. 123). This is because the contest of diverse opinions, in view of available evidence, is fundamental to the generation of knowledge, which is always fallible and thus prone to enhancement or transformation through meaningful exchange. Such exchange, moreover, implies detachment, tolerance, professionalism, and civility in the face of pluralism and ambiguity, all of which in turn entails forswearing the right to claim “that your god, your experience, your intuition, or your group is epistemologically privileged” (p. 119).

## 6 Problems with traditional free speech

We sympathize with these arguments for freedom of speech. Its advantages over both forms of safetyism and degraded free speech seem clear. At the very least, freedom of speech demands of people a level of courage, resilience, and maturity that far surpasses the infantilism, paternalism, and dogmatism that totalizing and expressive safetyism breed along with dismissive intransigence and polarized alienation, the other two core features of wilful incommensurability.

The question we put forward for further research, however, is whether freedom of speech, as traditionally revered, is the solution. Is it sufficient for reversing the degenerations of democracy that so many have identified? Does it, for example, allow for the flow of information necessary for citizens “to exercise their sovereign powers in an educated manner” (Bhagwat and Weinstein, 2021, p. 104)? Does it help to cultivate a polity in which citizens “see themselves as part of a shared political community—willing to accept their inevitable differences and accommodate governance within and across them” (Kramer, 2022, p. 26)? Further, does it, in the end, help to bring about the solidarity required to address the pressing existential crises that ominously loom before humanity? Does it actually encourage the legitimate comparison of differing perspectives in a way that empowers diverse individuals and groups to examine their own assumptions, adjust accordingly, and advance their own understandings? More bluntly, does it actually conduce to true freedom itself? That is, does it succeed in helping us transcend both forms of safetyism, dismissive intransigence, and polarized alienation? Or, on the contrary, does it invariably backfire, undermining its own objectives, leading once again to wilful incommensurability and degraded free speech?

Our point, to be clear, is not that freedom of speech is inherently problematic. And we are certainly not making a case for censorship. On the contrary, freedom of speech must be defended.

What we are suggesting is that, if we (all of us) are truly concerned with rejuvenating democracy and, specifically, with honoring Mill’s admonition that we must learn from each other for the good of both the individual and society—to avoid the spread of dead dogma—it would be *most productive to transform the way in which we practice free speech itself*. Without such a transformation, it is difficult to imagine how we can move past the alienation and disillusionment that weighs society down and compels so many of us to cocoon ourselves within our factions—from which, utilizing degraded free speech, we then criticize others with such contempt in the name of free speech. It is difficult to imagine how we can move past the wilful incommensurability we have fallen into, and hence the fragmentation and disillusionment that aspiring demagogues find so easy to exploit for their personal benefit.

Such a transformation, we propose, involves reimagining what many now consider to be natural features of freedom of speech. For example, advocates of free speech emphasize the centrality of persuasion and compromise. But this assumes that people are motivated to treat each other as rational beings and are rational in turn; that people are actually interested in hearing each other out and are open to compromise or being persuaded; that people with different perspectives and life experiences have a sufficiently comparable opportunity and ability to participate in the marketplace of ideas—which is obviously not the case since those with greater resources routinely impose their versions of the truth, effectively freezing out those with fewer resources from making their own contributions (Marshall, 2021, p. 55); and that people actually care about the truth. In order to get to the point where persuasion and compromise are even possible, we have to reckon with some fundamental dispositional and structural barriers. But even more than that: Should not free speech be aimed at something much more, such as mutual upliftment, solidarity, and expanding horizons of shared understanding that are as attuned as possible to reality as it is and the way it could become?

Advocates of free speech such as those referenced in this essay also argue in favor of offensive comedy and political satire. But how does such humor—feeding as it does on our reductionist cravings to see “the other” diminished—not end up reinforcing divisiveness and the walls of wilful incommensurability that need to be torn down? Certainly, a case can be made that people should be thick-skinned. But is that really the gist of the matter? Or is not such pointed comedy, far from changing hearts and minds for the better, instead hindering our ability to foster the very solidarity we need to address fundamental problems and flourish as a human species? These, it must be acknowledged, are involved questions that deserve much scrutiny since not all humor and satire are the same.

Yet, the same can be asked of how we express ourselves more generally and its relationship to harm. In the wake of the recent attack on Salman Rushdie, many wrote extolling freedom of speech. For example, the title of an article by Weiss (2022) was “We Ignored Salman Rushie’s Warning: Words are Not Violence. Violence is Violence”. Interestingly, two days after that, Figliuzzi (2022) wrote an article titled “Yet another person has died in defense of Trump’s lies. When will it end?”. So, on the one hand, there may be a legitimate distinction between words and violence, as Weiss, Haidt, Doyle, and many others argue. And there is truth to the idea that we should be able to “take it” in the face of “hurtful” words and be able to steel ourselves to argue rationally against such words. On this point, Pinker (2021) rhetorically asks: “If you have to silence people who disagree with you, does that mean you have no good arguments for why they are mistaken?” (p. 43). There is additionally truth to the idea that being able to exercise freedom of speech is essential to human dignity (Grimm, 2020).

On the other hand, is it not also the case that lying, conspiracy theories, backbiting, malicious character attacks, and other forms of vindictive language—all rationalized as instances of free speech—can cause harm? That they can also engender radicalization, which in turn leads to violence and, more broadly, undermines democracy? As Fisher (2022) reports, scholars have “found that, across topics or political ideologies, as the number of moral-emotional words in an article increased, commentors grew significantly likelier to threaten or incite violence against some perceived enemy, usually someone named in the article” (p. 156). A prominent example is the attack in October 2022 on the husband of the Speaker of the United States House of Representatives. This attacked was prompted by online and other portrayals of her. In the wake of the attack itself, some people responded with mocking jokes and trifling comments, causing many others to lament that civility and virtue have been eclipsed. To these others, all semblance of humanity, let alone dignity, had been lost, while moral-emotional hate speech was rewarded socially and politically.

Which brings us to a final question: Does not partisan politics itself invariably contribute to the pitfalls of the two forms of safetyism, dismissive intransigence, and polarized alienation, and hence the dire problems facing humanity? Certainly, there are sincere politicians with good intentions seeking to highlight the benefits of their platforms and the validity of their claims. They value honest debate of the issues. Yet many are far more intent on calling out their opponents and vying for the most newsworthy soundbite or the most likes on social media. And they find that the best way to win the battle of soundbites, or get the most likes, is to name call or land the best insults—to find the best way to ridicule or “slam” their rivals. The result is a defamation fest that can easily degenerate into a politics of vengeance where all limits—normative, legal, and otherwise—on ambition and the insistent self are contested. Meanwhile, the subtleties of the issues—those that affect real people—are submerged under a morass of partisan wrangling fueled by factious animosity. Is this how democracy is supposed to function? Instead, does not this practice end up suckling the very resentment that inspires the demagogue to rise up and claim the system is corrupt and that he or she is the panacea? And, regrettably, does not the demagogue—for all his or her faults and hypocrisies, him or herself often being one of the main culprits and fomenters of the problem—actually have a point about the system?

In short, we, the authors, recognize that freedom of speech is essential to social transformation and transcending the morass of the culture of wilful incommensurability. But our thesis is that the form that free speech currently takes—with its emphasis on contest, calling each other out, being thick-skinned, twisting truth, *etcetera*—ultimately backfires because it compounds the very problems it seeks to surmount. Most alarmingly, it disaffects, marginalizes, and consequently stifles the search for truth, which is so essential to dealing with crisis and promoting wellbeing more generally. It goads people into taking sides, into entrenching themselves within their bubbles, and so undermines the provision Mill regarded as essential to the discovery of truth, namely, pursuing an unremitting encounter with diversity for all it is worth. It, further, distances us from “the kind of sacrificial leadership—embodied in individuals and institutions, local and national—that could remind us of what we owe each other and that could point us to deeper sources that, even in our profound and interminable disagreements, still might bind us together in our communities, our nation, and our shared humanity” (Hunter, 2020).

## 7 A case for attentive free speech

Our suggestion for future exploration is that for speech to transcend the morass of wilful incommensurability and be truly free, it needs to transform as well. It requires a wholly new mode of discourse which is far more attuned to reason and buoyed by the virtues of courage, discernment, and reverence. Further, transforming speech in this way is essential for promoting solidarity and freedom itself. Without such a transformation, it is difficult to imagine how inclusive, participatory decision-making can be achieved, much less how democracy can grow and thrive. This position may at first glance seem naïve. However, we suggest that believing that liberal democracy—and, more broadly, humanity itself—can continue the way it has is even more credulous as evidenced by the environmental, economic, political, social, and international crises that accumulate largely because we are not working effectively together. Then again, it may be the crises themselves that finally compel us to make the necessary transformation.

We thus recommend for further study the proposal that for speech to be conducive to both individual and collective flourishing, it must become *attentive free speech*. In this regard, one concept that will need further exploration is what “free” actually means as it pertains to speech. Our thesis is that for speech to be truly free, it cannot be what is commonly thought of as free.

Briefly, there are similarities between the concepts of attentive free speech and civil discourse, although the latter can be conceptualized in different ways (Laden, 2019; Keith and Danisch, 2020; Barnes et al., 2023). In general, civil discourse emphasizes the importance of encouraging a culture of free expression and respectful, productive debate (Douglas et al., 2021). It repudiates shaming, *ad hominem* attacks, and snap-judging as communication strategies. It upholds instead a spirit of curiosity aimed at productive inquiry that results in compromise, consensus, and, where possible, shared understanding. Underpinning such inquiry are the virtues of politeness, fair-mindedness, patience, courage, and humility. Some also emphasize the capacity to engage in active, or deep listening (Schudson, 1997) and the importance of fostering a collaborative, or dialogical, process, where meaning can be cocreated (Longo and Shaffer, 2019, p. 21). Central to civic reasoning is the ability to think through issues “using rigorous inquiry skills and methods to weigh different points of view and examine available evidence…guided by respect for fundamental human rights” (Lee et al., 2021, p. 1). Such reasoning further involves taking account of relevant contextual knowledge as well as the disposition to “empathize with others and to listen to and consider contrasting points of view” (Lee et al., 2021, p. 4). Organizations such as the National Institute for Civil Discourse at the University of Arizona,^9^ grassroots endeavor’s such as Civil Dialog Initiative in Canada,^10^ and university initiatives such as the recent appointment of Randy Boyagoda as the University of Toronto’s provostial adviser on civil discourse,^11^ are working to put such principles into action.

We suggest that attentive free speech largely incorporates but also goes beyond what is called for in civil discourse. By being attentive, we mean, first, that free speech must be *discerning*, which is part of what makes it truly free. Far from being intransigent and entrenched, those who engage in attentive free speech aim to be perceptive, observant, heedful, exploratory, and judicious, conscious of our interdependence and our shared humanity. They strive to understand reality as it is in its complexity, to become attuned to it so that they can change it if required. They are adventurous and seek to grapple with ambiguity, which they recognize is an essential part of life and of being free (de Beauvoir, 1948). To this end, they recognize the value of employing reason, critical thinking, and weighing relevant evidence, experience, and contextual knowledge carefully. They also recognize that their assumptions and biases can both enable and constrain the investigation of truth. It follows that they are unreservedly oriented outwards because they understand that others, in their diversity, may have insights into a given matter that may not otherwise be readily apparent. There is always the potential to see more clearly and comprehensively in our diversity. We thus lose if we do not actively strive to discern it—if we do not venture beyond that which we find familiar; we sacrifice our freedom to know.

By being attentive, we also mean, second, that free speech must be *thoughtful*, *caring*, and *uplifting*, which are also essential to speech being free. Entailing more than just being civil, such speech can be described as incorporating a spiritual disposition toward others, even a reverence toward them. Those who engage in attentive speech aspire, at minimum, to be kind, sympathetic, and abundantly conscious of the comfort of others, and, to the extent possible, to create welcoming environments in which different perspectives can be readily shared. Cognizant that civil discourse can be used by those in power as an excuse to suppress the dissent of the oppressed (Barnes et al., 2023), they endeavor to be keenly alert to the subjective experience of others, to their underlying human susceptibilities, and particularly to the voices of those who have been historically marginalized. More than this, they conscientiously seek to mine different perspectives for points of unity upon which to build more expansive and harmonious understandings of the way things are and how they can advance. They carefully choose their words with the aim of elevating all participants, of eliciting their insights, of integrating their ideas where possible, and, in so doing, of collaboratively weeding out that which is fallacious, factious, or deleterious, thus achieving what Gadamer (2013) calls a fusion of horizons. In this way, they contribute to each other’s freedom to perceive, explore, and thrive, and hence their own freedom to do the same.

Those engaged in attentive speech thus embrace a mode of learning that is imbued with the virtues of humility, detachment, inquisitiveness, resilience, joyfulness, and *love*. They recognize along with Murdoch (2014) that freedom “is a function of the progressive attempt to see a particular object clearly” (p. 38), and that “[i]t is in the capacity to love, that is to *see*, that the liberation of the soul from fantasy consists” (p. 82); with Weil (2015) that “it is necessary to have a regime where the public expression of opinions is defined less by freedom and more by an atmosphere of silence and attention wherein this weak and inept cry can make itself heard” (p. 106); with Honneth (1995) that when reciprocal recognition is characterized by love, self-confidence becomes possible, and that such confidence, in turn, is a precondition for productive social relations, self-respect, and the constructive affirmation of one’s individuality; with Brooks (2023c) that “[t]here is one skill that lies at the heart of any healthy person, family, school, community organization, or society: the ability to see someone else deeply and make them feel seen—to accurately know another person, to let them feel valued, heard, and understood” (p. 9); with Weinberg (2011) that “[e]ffectively addressing the crises now disrupting human affairs will require new models of social transformation that…can emerge only from a fundamental change in consciousness about who we are [and] how we regard others who enter our ambit—no matter how near or distant” (pp. 77–78); with Benjamin (2022) that “a microvision of justice and generosity, love, and solidarity can have exponential effects” (p. 16); with the Akan maxims that “Life is mutual aid” and “Humanity has no boundary” (see Gyekye, 2010); with Indigenous philosophy that the teachings of humility, honesty, respect, courage, wisdom, truth, and love are sacred (Bouchard and Martin, 2016); with Nhat Hanh (2015), who is “committed to cultivating loving speech and deep listening in order to bring joy and happiness to others and relieve others of their suffering” (p. 84); and with Bahá’u’lláh (1988), who asserts: “Every word is endowed with a spirit”; that, consequently, “the speaker or expounder should carefully deliver his words at the appropriate time and place” recognizing that “One word is like unto springtime causing the tender saplings of the rose-garden of knowledge to become verdant and flourishing, while another word is even as a deadly poison”; and that it is therefore prudent to speak “with utmost leniency and forbearance so that the sweetness of [our] words may induce everyone to attain that which befitteth man’s station” (pp. 213–214). At core, this station is one of nobility. Our speech thus needs to reflect the inherent nobility of every human being, which is essential for the generation of mutually liberating knowledge.

Such insights are supported by research that highlight the impact that empathy, inclusivity, and trust have on innovation and prosocial outcomes related to both individual and group success and wellbeing (Heffernan, 2014; Teding van Berkout and Malouff, 2015; Clark et al., 2019). They have also inspired one of the authors of this paper and Karlberg to articulate the elements of what we call a *consultative epistemology*, the purpose of which is to enable proponents of different paradigms to, “with humility and detachment, better learn how to probe and sift through their respective truth claims, assess the relative merits of these claims, retain and integrate that which is relatively true to reality, and discard what is not” (Smith and Karlberg, 2009, pp. 92–93). This approach to epistemology is inspired by the Bahá’í concept of consultation (Karlberg, 2004, pp. 139–145; Smith and Ghaemmaghami, 2022) and has affinities with the approaches of thinkers mentioned in the previous paragraph as well as others such as Habermas (1984), Longino (1990), Freire (2000), Bohm (2004), Black (2015), hooks (2018), and Buber (2023). As with the case for a consultative epistemology, our case for attentive free speech is explicitly premised on the conviction that to genuinely advance, a fundamental transformation is required in how we (all of us) view ourselves in relationship to our own persons, to each other, to our communities, to our world, and to our individual freedom to express what we each sincerely think. Only in this way can we transcend the totalizing and expressive safetyism, dismissive intransigence, and polarized alienation that perpetuate the culture of wilful incommensurability and the degraded free speech associated with it.

## 8 Conclusion

In this paper, we have taken as our point of departure Calhoun et al.’s contention that liberal democracy is facing a crisis and that for it to evolve, it is essential to think big. For them, as well as for other observers, thinking big involves achieving an unprecedented level of social transformation aimed at cultivating solidarity, empowering citizen efficacy, and promoting the common good. We have argued specifically that a major challenge to democratic participation is the degraded way in which people speak to each other. Consequently, speech itself needs to transform. Otherwise, it will never be truly free nor advance the democratic project.

The main undertaking of this paper has been to articulate a framework for diagnosing the degraded state of public discourse. The core theme is that our liberal democracy has largely fallen into a condition of wilful incommensurability, which features both totalizing and expressive safetyism, dismissive intransigence, and polarized alienation. As pointed out in Section 2, there are notable variations and exceptions to this condition; we are not all equally under its spell. In general, however, the public sphere is now characterized by a pervasive, if not an unmitigated, aversion to reach out, identify points of unity, benefit from diverse perspectives, and discover truth in all its potential complexity. The type of speech associated with wilful incommensurability we have called degraded free speech. Such speech is degraded because, while it is habitually unrestrained, it is often so in service of indulging the safetyist need to shelter under the wing of simplicity and homogeneity, safe from having to deal with complexity and the challenge of perceived otherness. It presumes fragility in the face of alleged threat, both on the part of the individual and the group being challenged. This degraded condition of free speech, moreover, has been exacerbated by the toxic influence of social media, particularly over the last decade or more.

With this framework for diagnosing the present condition of speech in mind, we then articulated our sympathy for the position of those who argue on behalf of freedom of speech as traditionally understood. However, we raised questions about whether or not traditional free speech is up to the job of vitalizing a democracy that is flailing, of adequately grappling with the many existential crises pressing in upon humanity, and, more generally, of adhering to Mill’s counsel that people should continuously subject their ideas to diversity of thought to avoid succumbing to dogmatism. More specifically, we raised questions about its capacity to withstand the pull of both forms of safetyism, dismissive intransigence, and us-versus-them, or polarized, alienation. While traditional free speech may—that is, when civility holds sway—allow for persuasion and the force of the better argument to prevail, it is also at risk of inducing resentment, ill will, and ultimately the very safetyism, intransigence, and polarization that free speech advocates understandably find so disagreeable.

In view of these observations, we suggested for future research the thesis that the transformation called for by Calhoun et al. and others must entail an embrace of attentive free speech, which has some similarities with civil discourse, but which is specifically characterized by both discernment and thoughtfulness entailing a disposition toward mutual upliftment grounded in love. Our thesis is that attentive free speech is essential for achieving what many free speech advocates champion, including more participatory societies that are increasingly “accountable and responsive to people who aren’t yet in the room” (Táíwò, 2022, p. 116) and that simultaneously promote a spirit of exploration, solidarity, wellbeing, and freedom itself. The overall goal of attentive free speech is to enable all of us to become “vectors of justice, spreaders of joy, transforming our world so that everyone has a chance to thrive” (Benjamin, 2022, p. 16). It is to transcend wilful incommensurability and degraded free speech, and thus to achieve an evolving unity in diversity of understanding that, in turn, helps to foster the solidarity required to transform democratic decision making, address crises, and cultivate a state of freedom that is reciprocally, and collectively, energizing and elevating. Such freedom, we propose, is both true freedom and true safety.

As acknowledged, some may view our proposal as questionable, even naïve. And there is undeniably much to consider. For example, we have not in this paper directly addressed the relationships between power, elitism, prejudice, and discourse. That needs to be done in future work. It will also be necessary to articulate how attentive free speech can be operationalized and elevate democratic discourse in practice. But we suggest that the presumption that liberal democracy and its current mode of speech can fruitfully continue as they have is even more implausible. Given the growing challenges facing humanity, it seems timely and pragmatic to consider a new approach to how we speak and work with one another to address those challenges. To this end, we have cited some research and a sampling of thinkers from various traditions that help point the way.

## Data availability statement

The original contributions presented in the study are included in the article/supplementary material, further inquiries can be directed to the corresponding author.

## Author contributions

All authors listed have made a substantial, direct, and intellectual contribution to the work and approved it for publication.

## Footnotes

^1^By *clercs*, Applebaum is drawing upon the French essayist Julien Brenda’s book *La trahison des clercs*, in which, as she explains it, he accused “both far-right and far-left ideologues…of betraying the central task of the intellectual, the search for truth, in favor of particular political causes. Sarcastically, he called these fallen intellectuals *clercs*…” (p. 17).

^2^It is important to note that much that can be inferred from the data that cannot be explored here, including the finding that the climate of free speech varies significantly across college campuses.

^3^It is also worth noting that expressive safetyism can perversely contribute to the further dissemination and deepening of the very views it objects to. Speakers in this climate can book speaking appearances on campuses anticipating that they will be shouted down or canceled, and then use this as further evidence that they are the marginalized ones, heroically speaking truth to power. Given that no meaningful censorship is possible on the internet, their ideas still get out.

^4^As noted below in relationship to attentive free speech, some emotions, or emotional dispositions, such as love and compassion, can be conducive to rational thought.

^5^Plagued by this mindset, we also lose sight of the wisdom found in so many traditions—such as Buddhism, Stoicism, and recent psychological approaches stressing mindfulness and CBT—that the primary source of fear is how we think about threats, not necessarily the threats themselves (although, these can certainly play a role).

^6^We do not mean to suggest that people are never otherwise so wilfully inclined. See, for example, Heffernan (2011) for many examples of wilful blindness. We do maintain, however, that the inclination has become especially inordinate with the growing prevalence of safetyism.

^7^See for example Barak Obama on CNN at https://www.cnn.com/videos/politics/2023/06/23/obama-us-politics-amanpour-vpx.cnn.

^8^She distinguishes this from good or healthy conflict, which can lead somewhere useful and does not collapse into dehumanization.

^9^https://nicd.arizona.edu.

^10^http://civildialogue.ca.

^11^https://www.utoronto.ca/news/randy-boyagoda-appointed-u-t-s-provostial-adviser-civil-discourse.
